# Cardiometabolic biomarker patterns associated with cardiac MRI defined fibrosis and microvascular dysfunction in patients with heart failure with preserved ejection fraction

**DOI:** 10.3389/fcvm.2024.1334226

**Published:** 2024-03-04

**Authors:** Connor Siggins, Jonathan A. Pan, Adrián I. Löffler, Yang Yang, Peter W. Shaw, Pelbreton C. Balfour, Frederick H. Epstein, Li-Ming Gan, Christopher M. Kramer, Ellen C. Keeley, Michael Salerno

**Affiliations:** ^1^ Department of Chemistry, University of Virginia, Charlottesville, VA, United States; ^2^ Cardiovascular Division, Department of Medicine, University of Virginia Health System, Charlottesville, VA, United States; ^3^ UCHealth Heart and Vascular Clinic, Greeley Medical Center, Greeley, CO, United States; ^4^ BioMedical Engineering and Imaging Institute, Icahn School of Medicine at Mount Sinai, New York, NY, United States; ^5^ New England Heart and Vascular Institute, Catholic Medical Center, Manchester, NH, United States; ^6^ Baptist Heart & Vascular Institute, Baptist Health Care, Pensacola, FL, United States; ^7^ Department of Biomedical Engineering, University of Virginia, Charlottesville, VA, United States; ^8^ Department of Molecular and Clinical Medicine, Institute of Medicine, Sahlgrenska Academy at the University of Gothenburg, Gothenburg, Sweden; ^9^ Department of Radiology and Medical Imaging, University of Virginia Health System, Charlottesville, VA, United States; ^10^ Department of Medicine, University of Florida, Gainesville, FL, United States; ^11^ Division of Cardiovascular Medicine, University of Florida, Gainesville, FL, United States; ^12^ Department of Radiology, Stanford University, Stanford, CA, United States; ^13^ Department of Medicine, Cardiovascular Medicine, Stanford University, Stanford, CA, United States

**Keywords:** heart failure with preserved ejection fraction, biomarkers, perfusion, extracellular volume, cardiometabolic

## Abstract

**Introduction:**

Heart failure with preserved ejection fraction (HFpEF) is a complex disease process influenced by metabolic disorders, systemic inflammation, myocardial fibrosis, and microvascular dysfunction. The goal of our study is to identify potential relationships between plasma biomarkers and cardiac magnetic resonance (CMR) imaging markers in patients with HFpEF.

**Methods:**

Nineteen subjects with HFpEF and 15 age-matched healthy controls were enrolled and underwent multiparametric CMR and plasma biomarker analysis using the Olink® Cardiometabolic Panel (Olink Proteomics, Uppsala, Sweden). Partial least squares discriminant analysis (PLS-DA) was used to characterize CMR and biomarker variables that differentiate the subject groups into two principal components. Orthogonal projection to latent structures by partial least squares (OPLS) analysis was used to identify biomarker patterns that correlate with myocardial perfusion reserve (MPR) and extracellular volume (ECV) mapping.

**Results:**

A PLS-DA could differentiate between HFpEF and normal controls with two significant components explaining 79% (Q2 = 0.47) of the differences. For OPLS, there were 7 biomarkers that significantly correlated with ECV (R2 = 0.85, Q = 0.53) and 6 biomarkers that significantly correlated with MPR (R2 = 0.92, Q2 = 0.32). Only 1 biomarker significantly correlated with both ECV and MPR.

**Discussion:**

Patients with HFpEF have unique imaging and biomarker patterns that suggest mechanisms associated with metabolic disease, inflammation, fibrosis and microvascular dysfunction.

## Introduction

Heart failure with preserved ejection fraction (HFpEF) accounts for about 50% of heart failure cases (1). Furthermore, HFpEF is more common in older patients and in women and has a 5-year mortality rate of up to 56% (1). HFpEF is often used as a broad term to describe a heterogenous group of patients with multiple associated comorbidities and etiologies that share a similar clinical presentation in patients with preserved ejection fractions. Although the mechanism of HFpEF is not fully understood, it is widely recognized as a complex disease process that is heavily influenced by metabolic disorders, systemic inflammation, myocardial fibrosis, microvascular dysfunction, and interactions with other organ systems (2). Many pharmacologic therapies used to prevent adverse remodeling in patients with reduced ejection fraction have not demonstrated improved outcomes in HFpEF. To date only studies of sodium-glucose-co-transporter 2 (SGLT2) inhibitors and mineralocorticoid receptor antagonists (MRA) have demonstrated a reduced risk of adverse outcomes in HFpEF (3). Although the mechanism of MRAs are well studied, SGLT2 inhibitors are a novel drug with several metabolic, hemodynamic, and organ-specific effects that are not well understood. Additional research is needed to characterize the pathways that contribute to HFpEF and identify potential therapeutic targets.

Cardiac magnetic resonance (CMR) imaging has been shown to be a useful modality for evaluating myocardial perfusion, strain, and fibrosis (4). Extracellular volume (ECV) mapping is a CMR technique that measures interstitial volume fraction of the myocardium by measuring the T1 relaxation times of the myocardium and blood pool before and after gadolinium contrast. Increased ECV is associated with diffused fibrosis in the absence of focal scar (5). Myocardial perfusion reserve (MPR) can be used to detect microvascular dysfunction in patients without obstructive coronary disease. MPR is calculated based on the ratio of stress and rest perfusion during quantitative first-pass perfusion imaging (6). Our prior findings showed that patients with HFpEF have higher prevalence of diffuse fibrosis and coronary microvascular dysfunction based on CMR (4), which are both independently associated with adverse outcomes (7–10). These imaging markers may provide a clue to key molecular pathways that lead to the development of HFpEF and serve as surrogate measurements of pharmacologic treatment response. In our present study, we analyze how CMR imaging markers relate to cardiometabolic plasma biomarkers in HFpEF.

In a prior study, we investigated associations between ECV and biomarkers of inflammation in hypertensive heart disease (11). We used dimensionality reducing statistical methods to analyze correlations between ECV and left ventricular mass index (LVMI) and cardiovascular plasma biomarkers in hypertensive patients with and without left ventricular hypertrophy and healthy controls. The present study utilizes similar methodology to analyze associations between ECV, MPR, and cardiometabolic plasma biomarkers in a distinct cohort of HFpEF patients and healthy controls.

## Methods

### Enrollment

Subjects with HFpEF and age-matched controls were prospectively enrolled for this IRB approved clinical research study. There were two prespecified aims for this study: (1) Compare echocardiography, cardiopulmonary exercise testing, and CMR findings, and (2) Identify relationships between CMR and cardiometabolic biomarkers. The results of the aim 1 have been previously published (12). The current study reports the findings from the second aim. Subjects met HFpEF inclusion criteria if they were age 18–85, had New York Heart Association (NYHA) classification ≥ II or B-type natriuretic peptide (BNP) ≥ 150 pg/ml, ejection fraction (EF) > 45%, and had at least an echocardiogram with grade 1 diastolic dysfunction or a right heart catheterization with an elevated pulmonary capillary wedge pressure. Exclusion criteria were secondary hypertension, prior myocardial infarction, severe valvular disease, pericardial disease, infiltrative cardiomyopathy such as sarcoidosis or amyloidosis, hypertrophic cardiomyopathy, idiopathic pulmonary artery hypertension, or heart failure with reduced left ventricular (LV) ejection fraction with subsequent recovery of function. Subjects with contraindications to CMR such as metallic implants, severe claustrophobia, pacemakers/defibrillators, and estimated glomerular filtration rate <45 ml/min/1.73 m^2^ were also excluded. History of persistent atrial fibrillation with rapid ventricular rates >100 bpm were excluded due to poor image quality. Fifteen age-matched normal controls, who were free of cardiovascular disease were prospectively enrolled for comparison.

The study protocol included a history and physical exam, cardiopulmonary exercise testing, transthoracic echocardiogram, CMR, and inflammatory biomarkers. The patients medical record was reviewed for other recent laboratory results.

### Biomarkers

Venipuncture was used to collect 20 ccs of blood from patients during their initial evaluation in which a medical history and physical exam was conducted prior to CMR. Blood samples were heparinized and insoluble impurities were removed by centrifuge. Plasma biomarkers were analyzed using the Olink® Cardiometabolic Panel (Olink Proteomics, Uppsala, Sweden), which simultaneously measures 92 cardiometabolic disease-related human proteins in Normalized Protein eXpression (NPX) units in a Log2 scale. The reagent kit uses proximity extension assay in which antibody probe pairs bind with target proteins in the sample. A polymerase chain reaction (PCR) reporter sequence was created and subsequently quantified with real-time PCR. Additional information including validation can be found at http://www.olink.com.

### CMR protocol

CMR studies were performed on a scanner at 1.5 Tesla (Aera or Avanto, Siemens Healthineers, Erlangen, Germany). Scout imaging was performed followed by balanced steady-state free precession cine sequences to measure LV volumes, mass, and function. A short-axis stack of cine images with 8 mm thickness and 2 mm gap was acquired for the entire LV. Three long-axis images in the 2, 3, and 4-chamber views were obtained. Circumferential and radial strain were acquired using spiral cine displacement encoding with stimulated echoes (DENSE) pulse sequence (13, 14). T1 mapping images were obtained at the base- and mid-ventricular short-axis with a modified Look-Locker inversion recovery (MOLLI) sequence prior to contrast administration, at 5 and 10 min after rest perfusion imaging, and at 5, 10, and 15 min after stress perfusion imaging.

For stress perfusion imaging, adenosine was given at 140 *μ*g/kg per minute through peripheral intravenous access over 3–4 min. Three short-axis slices were acquired over 60 heart beats during an intravenous bolus of 0.075 mmol/kg at a rate of 4 ml/s by power injector with Magnevist gadolinium contrast (Bayer Healthcare, Whippany, New Jersey, USA). Rest perfusion was performed 15 min after stress imaging. First-pass quantitative perfusion completed using a dual-sequence approach with a vendor-provided work in progress (WIP) package. A WIP package is a pulse sequence in development that is made available for research. Low-resolution arterial input function (AIF) image and 3 myocardial tissue function (TF) images were acquired at every RR interval with a saturation-recovery gradient echo pulse sequence. AIF and TF proton density weighted images were acquired to correct for surface-coil related intensity inhomogeneity. Signal intensity was converted to gadolinium concentration units using Bloch simulation modeling as previously described (15, 16). Late gadolinium enhancement (LGE) with phase-sensitive inversion recovery pulse sequences were obtained at 5 min after resting perfusion based on the standard SCMR guideline protocols (17).

Cine images were analyzed by an experienced user with QMASS (Medis Medical Imaging Systems, Leiden, the Netherlands). Cine images were segmented at short-axis each slice location to measure LV end-diastolic volume, end-systolic volume, stroke volume, EF, and myocardial mass.

Perfusion quantification was performed with the constrained Fermi function deconvolution method on a pixel-wise basis in MATLAB (Mathworks, Natick, Massachusetts, USA) (18). MPR was calculated for each patient as a ratio of the stress perfusion to rest perfusion.

Using previously described methods (19), average peak systolic circumferential strain (Average Circ S_S_), average peak systolic circumferential strain rate (Average Circ S_SR_), and average peak early diastolic circumferential strain rate (Average Circ e'_SR_) were computed offline from DENSE images with a custom MATLAB script. Base and mid slices were used for measurements (strain is a unitless value).

### Statistical analysis

Continuous variables are reported as their mean and standard deviation. Categorical values are reported as percentages and number. Subject groups were compared with Wilcoxon rank-sum test or Fisher's Exact test for continuous and categorical values respectively. Significance was defined as *p*-value < 0.05.

To address the multicollinearity given that there were more variables than subjects, variations of principal component analyses were used. The statistical methods employed in this study are similar to our prior study looking at the relationship between CMR and biomarkers in patients with hypertensive heart disease (11). Importantly, there is no overlap in the cohort of patients or controls from that study and the present study; in the present study all patients underwent stress CMR studies, and a different biomarker panel was used. Differences between categorical subject groups were assessed using partial least squares discriminant analysis (PLS-DA) in two principal components using imaging and biomarkers. To identify the correlation between biomarkers and specific CMR parameters, orthogonal projection to latent structures by partial least squares (OPLS) analysis was implemented. For each OPLS model, loading values were calculated for the plasma biomarker variables with respect to a predicted CMR variable. The loading values were normalized to generate correlation coefficient estimates, given that the loading values are covariates of the predicted variable. Full jack-knife cross-validation was performed to determine the robustness of the model (Q2) and the contribution of each of the variables to the model. PLS-DA and OPLS analysis were performed with the ropls packages in R 4.0.3 (R Foundation for Statistical Computing, Vienna, Austria).

## Results

### Baseline characteristics

A total of 34 patients were included in the present study, 19 of whom had HFpEF. One patient was excluded given evidence of athletic remodeling based on history, echocardiography, and cardiopulmonary exercise testing. The final data set consisted of 15 controls and 18 HFpEF patients. Table 1 summarizes the demographics and clinical characteristics for the HFpEF and control groups. The average age of controls and HFpEF patients were 59 and 64 years respectively. There was a higher proportion of women in the HFpEF group, and the average body mass index (BMI) of the HFpEF group was significantly higher than that of the control group. Additionally, HFpEF patients were more likely to be taking medications for common comorbidities including type 2 diabetes and dyslipidemia.

**Table 1:** Baseline clinical characteristics.

	Controls (*n* = 15)	HFpEF (*n* = 18)	*p*-value
Age (years)	59 ± 9	64 ± 11	0.394
BMI (kg/m^2^)	27 ± 4	37 ± 7	<0.001
Male (%)	53 (8)	33 (6)	0.493
AA (%)	0 (0)	28 (5)	0.049
HTN (%)	20 (3)	83 (15)	<0.001
HLD (%)	13 (2)	72 (13)	0.001
DM (%)	0 (0)	61 (11)	<0.001
Current Smoker (%)	0 (0)	11 (2)	0.489
Former Smoker (%)	0 (0)	44 (8)	0.004
Pack Years	0 ± 0	10 ± 13	0.005
Aspirin (%)	27 (4)	50 (9)	0.284
Statin (%)	13 (2)	67 (12)	0.004
BB (%)	0 (0)	78 (14)	1.000
ACE/ARB (%)	20 (3)	72 (13)	0.005
CCB (%)	0 (0)	17 (3)	0.233
Diuretic (%)	7 (1)	94 (17)	<0.001
Insulin (%)	7 (1)	28 (5)	0.186
LV EF (%)	65 ± 5	62 ± 9	0.450
LV EDV (ml)	126 ± 34	145 ± 31	0.174
LV ESV (ml)	46 ± 11	55 ± 10	0.326
LV Mass (g)	93 ± 29	100 ± 36	0.706

Data is presented as mean ± standard deviation for continuous values or percentage (number) for categorical values.

Subject groups were compared with Wilcoxon rank-sum test or Fisher's Exact test for continuous and categorical values respectively. Significance was defined as *p*-value < 0.05.

AA, African American; ACE, angiotensin converting enzyme; ARB, angiotensin receptor blocker; BB, beta-blocker; BMI, body mass index; CCB, calcium channel blocker; DM, diabetes mellitus; EF, ejection fraction; EDV, end-diastolic volume; ESV, end-systolic volume; HFpEF, heart failure with preserved ejection fraction; HLD, hyperlipidemia; HTN, hypertension; LV, left ventricle.

### Imaging and biomarker pattern in HFpEF

**Figure 1:**
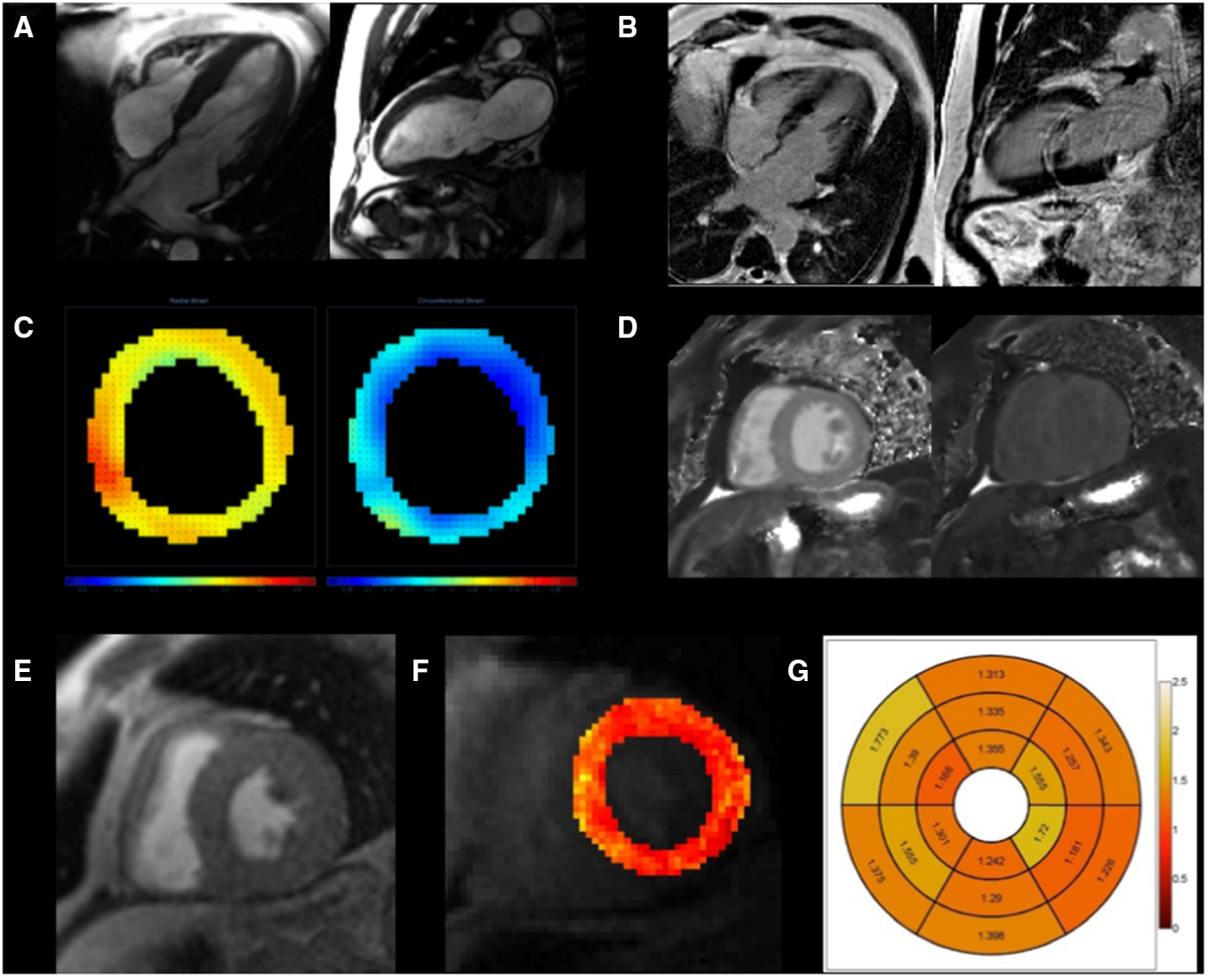
Multiparametric CMR imaging in a patient with HFpEF. Parameters include cine to measure function, LV mass, and LV volume (**A**), late gadolinium enhancement to identify focal fibrosis (**B**), radial (orange) and circumferential (blue) systolic strain with DENSE (**C**), T1 mapping to identify diffuse fibrosis (**D**), first-pass perfusion imaging at stress (**E**) with quantitative analysis (**F**) and associated bulls-eye plot of myocardial blood flows (**G**) Except for ECV and MPR, all other parameters were similar between HFpEF and normal controls.

**Figure 2:**
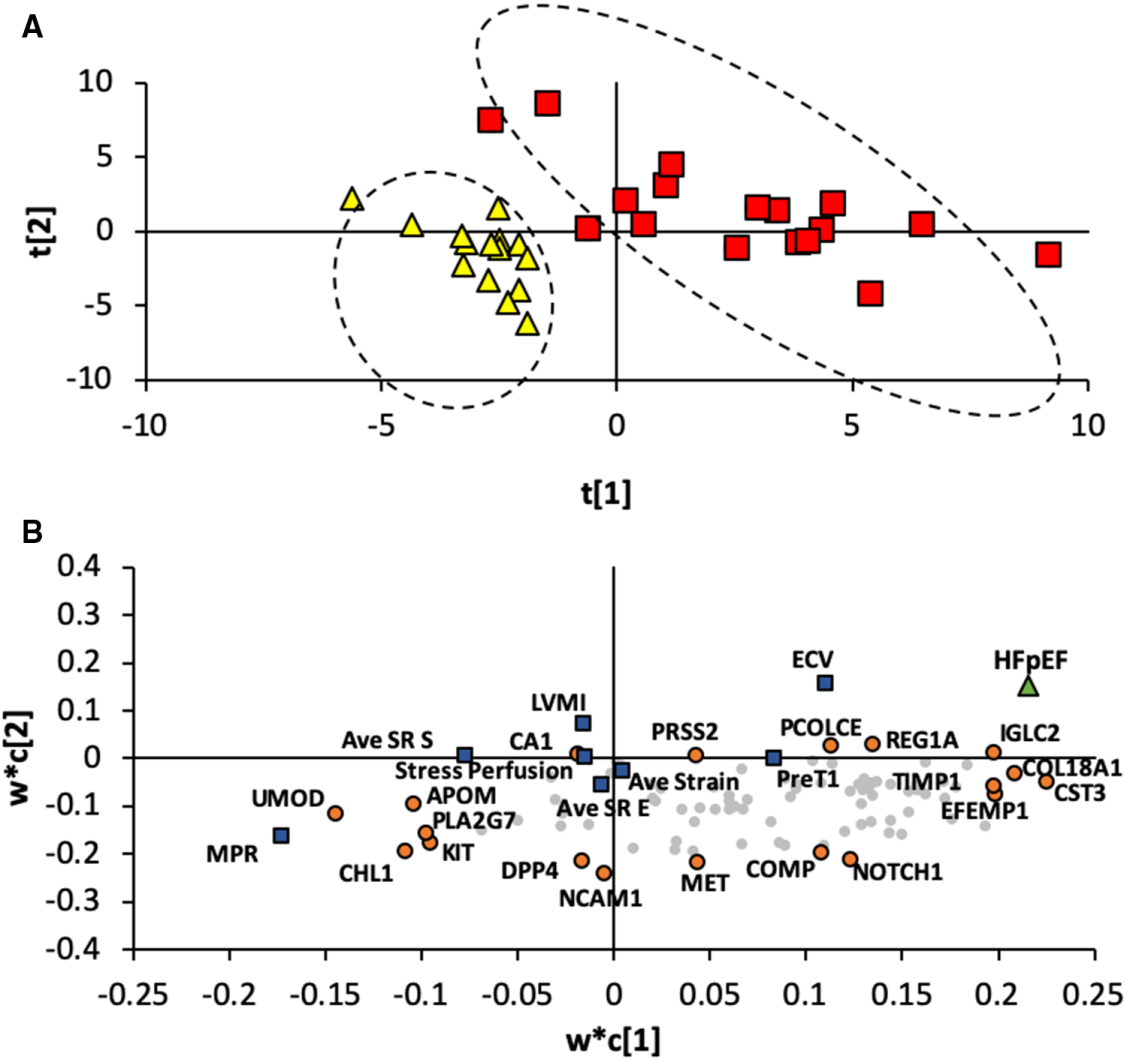
Results of the PLS-DA. (**A**) Individuals in the HFpEF and control groups plotted on two principle components. HFpEF patients and healthy controls are plotted as red squares and yellow triangles respectively. Ovals have been drawn to highlight the two groups. The two components in the PLS-DA explained 79% of the difference between the HFpEF and control groups. (**B**) Imaging and biomarkers plotted based on their weighting values in each component. Highly weighted imaging markers and biomarkers are plotted as blue squares and orange circles respectively. A projection of the HFpEF group is plotted as a green triangle. Markers closer to the projection are correlated with HFpEF.

A total of 8 imaging markers (Table 2) and 92 plasma biomarkers (Supplementary Table S1) were combined for the 33 patients in this analysis. Figure 1 shows an example of CMR parameters acquired for a subject with HFpEF. A PLS-DA generated two significant components explaining 79% (Q2 = 0.471) of the differences between normal and HFpEF subjects. Figure 2A shows the clustering of individuals based on the variables shown in Figure 2B. Normal subjects tended to have negative scores in the first (X-axis) and second (Y-axis) component. The most descriptive markers in the first component were Tissue inhibitor of matrix metalloproteinases (TIMP)1, Collagen type XVIII Alpha (COL18A)1, and EGF-containing fibulin-like extracellular matrix protein (EFEMP)1. In the second component, the most descriptive markers included Neural cell adhesion molecule (NCAM)1, Mesenchymal epithelial transition factor (MET), and Dipeptidyl-peptidase (DPP)-4. The most descriptive imaging markers were MPR and ECV for the first and second component respectively.

**Table 2:** CMR imaging markers.

	Controls (*n* = 15)	HFpEF (*n* = 18)	*p*-value
LVMI (g/m^2^)	47 ± 13	47 ± 16	0.711
Pre-T1 (ms)	1,025 ± 43	1,033 ± 77	0.890
ECV	0.26 ± 0.04	0.29 ± 0.04	0.020
Average Circ S_SR_ (s^−1^)	−0.96 ± 0.16	−1.04 ± 0.20	0.369
Average Circ e'_SR_ (s^−1^)	1.39 ± 0.44	1.36 ± 0.62	0.554
Average Circ S_S_ (unitless)	−0.16 ± 0.03	−0.16 ± 0.04	0.878
Global MPR (unitless)	3.42 ± 0.61	2.20 ± 0.48	<0.001
Stress Perfusion (ml/g/min)	1.83 ± 0.52	1.79 ± 0.06	1.000

Data is presented as mean ± standard deviation.

Subject groups were compared with Wilcoxon rank-sum test. Significance was defined as *p*-value < 0.05.

Circ, circumferential; e'_SR_, peak early diastolic strain rate; ECV, extracellular volume; HFpEF, heart failure with preserved ejection fraction; LV, left ventricular; MI, mass index; MPR, myocardial perfusion reserve; S_S_, peak systolic strain; S_SR_, peak systolic strain rate.

**Figure 3:**
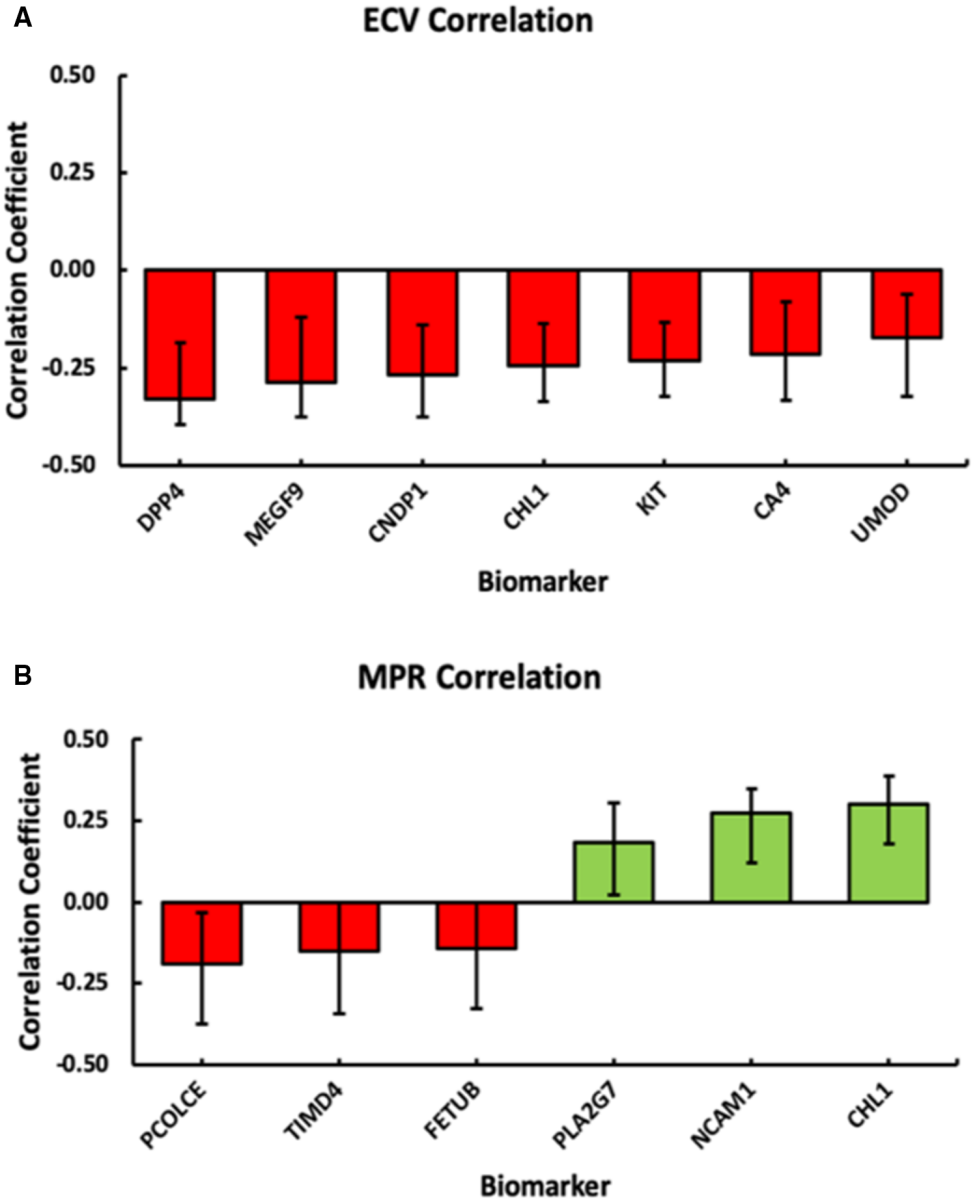
Estimated correlation coefficients for significant plasma biomarkers in the OPLS models for ECV (**A**) and MPR (**B**). Biomarkers with negative correlations are colored in red and those with positive correlations are colored in green. CHL1 was significantly correlated with both ECV and MPR. Error bars represent 95% confidence intervals after jackknife cross validation.

**Figure 4:**
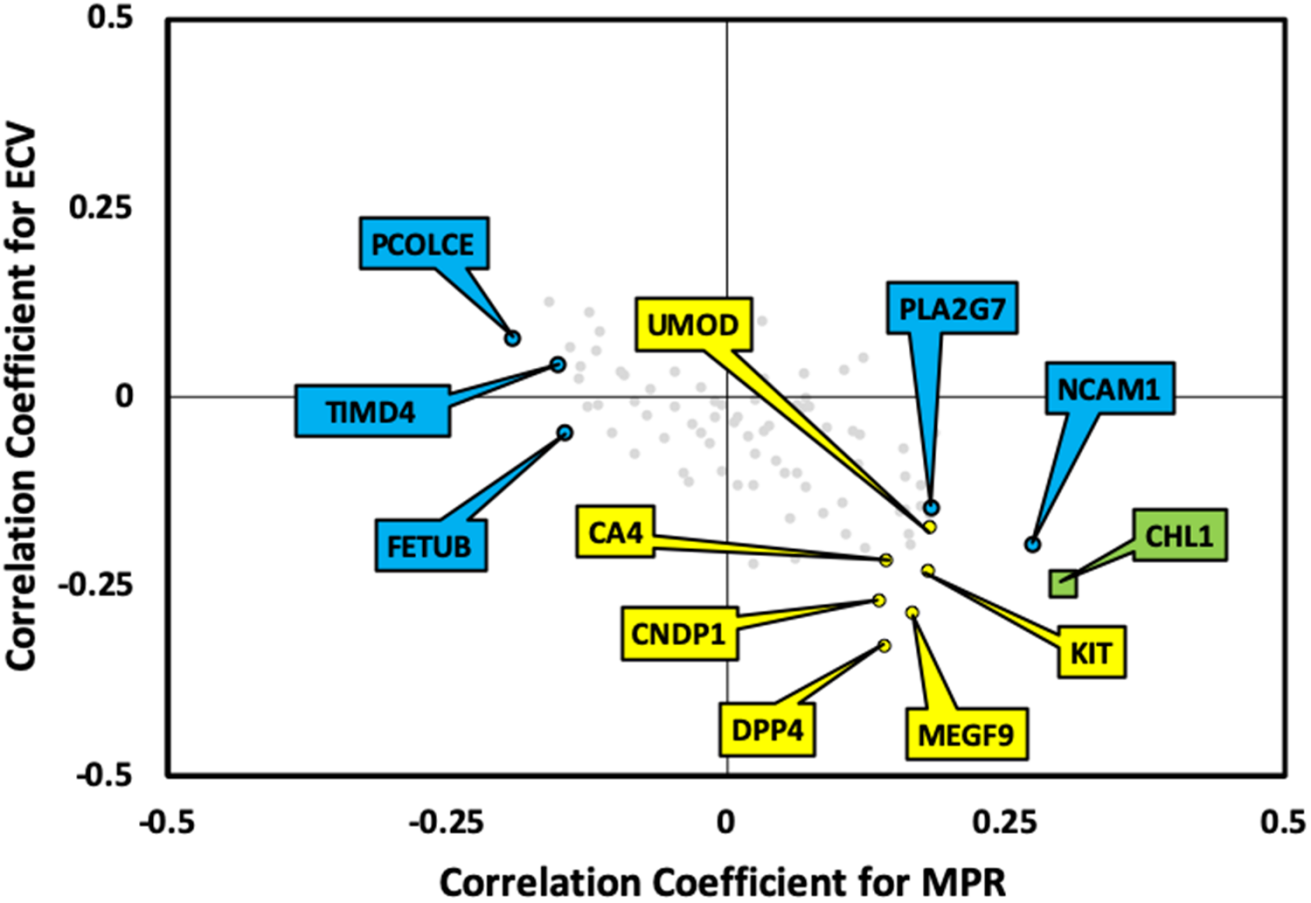
The relationship between biomarker patterns predicting myocardial perfusion reserve (MPR) and extracellular volume (ECV). The graph shows the correlation coefficients for the different biomarkers in the OPLS-model predicting MPR (X-axis) vs. the model predicting ECV (Y-axis). The blue labelled biomarkers correlate significantly with MPR, and the yellow labelled biomarkers correlate significantly with ECV. CHL1, labelled in green correlated with both imaging markers.

To identify biomarkers predicting ECV and MPR, respectively, two OPLS models from the 92 plasma biomarker variables were generated on the basis of individuals from both groups. Two patients were excluded from the OPLS analysis due to missing CMR data. The ECV model explained 85.3% (Q2 = 0.533) of the ECV data. The MPR model explained 92.4% (Q2 = 0.321) of the MPR data. Figure 3A illustrates significant correlation coefficient estimates in the model for predicting ECV. Figure 3B shows the corresponding data for MPR. Correlation coefficients estimates from each of the 92 biomarkers for ECV and MPR can be seen in Supplementary Table S1. Based on the two models, a total of 7 and 6 markers were found to be significantly correlated with ECV and MPR, respectively. Out of these, one was shared: Cell adhesion molecule (CHL)1. Figure 4 shows the combined correlations between the biomarker patterns predicting ECV and MPR.

## Discussion

In the present study, we used principal component analysis to identify correlations between plasma biomarkers from the Olink® Cardiometabolic Panel and CMR imaging markers in patients with HFpEF. Our findings may provide insight into the pathophysiology of HFpEF and potential targeted therapies. Below we review the literature regarding the plasma biomarkers included this study and their involvement in tissue remodeling and inflammatory pathways in the development of cardiometabolic syndromes.

### Markers associated with HFpEF

Based on the PLS-DA, ECV and MPR are important in distinguishing HFpEF patients from healthy controls. ECV serves as a marker of diffuse fibrosis and has been shown to be an independent predictor of cardiovascular events (9). MPR can be used to identify microvascular dysfunction in patients with non-obstructive coronary disease. Furthermore, it is associated with diastolic dysfunction and adverse events in patients with HFpEF (10). We previously showed that HFpEF patients have a high prevalence of diffuse fibrosis and coronary microvascular dysfunction (12). Our current findings showed that specific plasma biomarkers had high loading values and were therefore correlated with HFpEF. For the purpose of the present study, plasma biomarkers were correlated with ECV and MPR to identify potential associations with diffuse fibrosis and microvascular dysfunction respectively.

### Biomarkers correlated with ECV

Biomarkers that correlate with ECV demonstrate an association with metabolic disease, renal disease, and response to inflammation and ischemia. DPP-4 is a member of the peptidase SB9 family and helps to regulate insulin levels by degrading glucagon-like peptide (GLP)-1 and gastric inhibitory polypeptide (GIP), which stimulate the production of insulin by beta-cells in the pancreas. Two classes of drugs, DPP-4 inhibitors and GLP-1 agonists target this pathway and are used in the treatment of type 2 diabetes, a common comorbidity in heart failure patients. GLP-1 receptor agonists have shown a cardiovascular mortality benefit in type 2 diabetes and are an effective therapy for weight loss (20). A recent systematic review highlighted the beneficial effects of GLP-1 agonists on LV diastolic function in patients with type 2 diabetes as well as in mice with HFpEF (3). Studies of the effects of DPP-4 inhibitors on LV diastolic function and risk of hospitalization due to heart failure have yielded mixed results and vary between drugs. However, an animal study found that DPP-4 inhibition reduced the progression of cardiac fibrosis (21). It is clear that an epidemiological relationship exists between metabolic disease and cardiovascular disease. SGLT2 inhibitors have been shown to be effective in the treatment of HFpEF and has been approved for use by the FDA (22). There are many proposed mechanisms of action, but the primary pathway in which SGLT2 inhibitors mitigate heart failure exacerbations remains unknown. Continued research focused on identifying associations between diabetes and HFpEF is essential for targeted therapies.

Extensive research has documented the interaction between renal and cardiovascular function. Renal dysfunction is an important factor contributing to adverse remodeling of the myocardium. Uromodulin (UMOD) and Serum Carnosinase (CNDP)1 are both involved in impaired renal function. UMOD is a glycoprotein found in the epithelium of the thick ascending loop of Henle. Higher UMOD levels have been found to be associated with lower mortality from cardiovascular disease in older patients (23). Additionally, different polymorphisms of UMOD have been shown to contribute to cardiorenal function in patients with hypertension and cardiovascular disease (24). CNDP1 is a member of the peptidase M20A family and is responsible for the cleavage of carnosine. CNDP1 is a marker for diabetic nephropathy and cardiovascular mortality in female patients with type 2 diabetes mellitus (25).

Fibrosis is characterized by an accumulation of extracellular matrix, and occurs as part of an adverse response to myocardial injury. Many mechanisms may contribute to tissue damage including inflammation and ischemia (7). Mast/stem cell growth factor receptor Kit (KIT), and Carbonic anhydrase (CA)4 may be related to chronic inflammation or ischemia leading to myocardial injury and adverse remodeling. KIT is part of the tyrosine kinase family and is involved in cell signaling in a variety of processes. Notably, c-KIT positive cells have been investigated for their role in repairing damage to the myocardium (26). Additionally, tyrosine kinase inhibitors are class of drugs that inhibit the growth of tumors, but have the side effect of increased risk of adverse cardiac events including LV dysfunction and heart failure. Disruption of kinase pathways that inhibit the growth of tumors may also adversely affect other cell classes, including cardiomyocytes (27). Carbonic anhydrases catalyze the formation of bicarbonate from carbon dioxide and are important in a variety of physiological processes that involve pH regulation. CA4 is expressed in the membranes of epithelial and endothelial cells in various tissues. Studies investigating activity and expression of carbonic anhydrases in cardiovascular disease have found elevated CA1 and CA2 expression in diabetic ischemic cardiomyopathy (28) as well as elevated expression of CA2 and CA4 in patients with hypertrophic hearts (29). The current body of literature suggests that changes in CA expression may influence cardiovascular disease through a mechanistic relationship between expression of carbonic anhydrases and pH dysregulation in the myocardium (28, 29).

### Biomarkers correlated with MPR

Roughly half of the biomarkers that correlated with MPR were related to systemic inflammation and response to myocardial injury and ischemia. T-cell immunoglobulin and mucin domain-containing protein (TIMD)4 and Platelet-activating factor (PLA) acetylhydrolase-7 are both involved in immune system signaling and mediating inflammation. TIMD4 is a member of the immunoglobin superfamily and is involved the regulation of macrophages and in the proliferation of T-cells. Research has found elevated levels of TIMD4 in patients after ischemic stroke (30). PLA acetylhydrolase-7 is a member of the lipase family and is responsible for deactivating PLA, an important proinflammatory signaling molecule (31). Studies investigating the association between plasma levels of PLA acetylhydrolases and cardiovascular disease have found that higher levels of PLA acetylhydrolase-7 lead to an increased risk of cardiovascular disease (32).

NCAM1 is named for its involvement in cell-cell adhesion of neurons, and it may play a role in cell signaling. Expression of NCAM1 has been investigated as a biomarker for coronary artery disease, and one study found that plasma NCAM1 levels were negatively correlated with coronary artery disease (33). Additionally, NCAM1 expression in the myocardium has been shown to be associated with worsened LV function and increased remodeling (34). The current body of literature indicates that NCAM1 may be involved in the cellular response to myocardial damage.

Fetuins are named for their role during fetal development, but research has found that Fetuin B (FETUB) is involved in dyslipidemia, metabolic disease, and insulin signaling in the heart. FETUB has been shown to be elevated in the livers of type 2 diabetes patients with non-alcoholic fatty liver disease as well as in patients with coronary artery disease (35). Studies in diabetic mice have shown that fetuin B inhibits cardiac insulin signaling by interacting with the insulin receptor-beta subunit leading to myocardial injury (36). Fetuin B may be one of many pathways that link metabolic disease with myocardial injury.

### Biomarkers correlated with both ECV and MPR

One biomarker, CHL1, correlated with both ECV and MPR. CHL1 is a member of the L1 family of cell adhesion molecules and is involved in cell signaling in a variety of pathways. CHL1 suppresses beta-cell proliferation by inhibiting the ERK pathway, and decreased expression of CHL1 has been shown to lead to the proliferation of beta-cells in mice consuming a high fat diet. CHL1 may be involved in compensatory hyperplasia of pancreatic beta-cells in pre-diabetes and metabolic syndrome (37). Metabolic syndrome and type 2 diabetes are well characterized comorbidities for cardiovascular disease, and disruption of metabolic pathways and processes has been shown to lead to adverse remodeling in the myocardium.

### Biomarkers without significant correlations

There were biomarkers included in the present study that did not correlate with either ECV or MPR despite expectations. Transforming growth factor beta receptor type 3 (TGFBR3), COL18A1, and TIMP1 have all been previously studied for their roles in cardiac fibrosis but did not correlate with ECV in our cohort (38–40). COL18A1 is a member of the multiplexin family. Studies of other collagen type proteins have found that during adverse cardiac remodeling in fibrosis, collagen derived peptides may be released into circulation and serve as potential biomarkers for fibrotic and inflammatory changes (39). TGFBR3 is involved in TGF-beta signaling, which plays a key role in cytokine regulation, cardiac remodeling after injury, and the development of fibrosis (40). TIMP1 is a member of the tissue inhibitors of metalloproteinases family. TIMPs 1–4 have all been shown to contribute to cardiac fibrosis through various mechanisms (38, 41). However, both TIMP1 and COL18A1 were associated with HFpEF in the PLS-DA model. Interestingly, TIMP1 and COL18A1 have also been shown to be associated with endothelial cell migration, angiogenesis, and microvascular function (42, 43). This may reflect the paradigm shift in our understanding of HFpEF pathophysiology; microvascular endothelial inflammation rather fibrosis is a key driver in the development of HFpEF. Although a completely different biomarker panel (Olink Proseek Multiplex CVD I^96 × 96^) was used in our prior hypertensive heart disease study (11), most of the biomarkers that correlated with ECV and left ventricular hypertrophy were associated with inflammatory pathways such as interleukin-18 (IL-18), interleukin-6 (IL-6), and tumor necrosis factor (TNF). These biomarkers were not included in the Olink® Cardiometabolic Panel used for the current study.

### Limitations

The associations demonstrated in the present study are correlational. As such, investigation of potential causes for the relationships observed in this study should be pursued in future research. An important limitation in this study is the small sample size. With the small sample and relatively large number of biomarkers, a version of principal component analysis was used as a dimensionality reducing technique. Additionally, conventional clinical labs such as hemoglobin A1c and cholesterol were not collected prospectively for this study. The effects of certain variables such as severity of HFpEF, comorbidities, or medications on imaging and plasma biomarkers could not be evaluated.

## Conclusion

ECV and MPR exhibit unique biomarker patterns that suggest the activity of different pathways associated with fibrosis and microvascular dysfunction in patients with HFpEF. Although the small sample size limits the degree to which conclusions can be drawn about individual biomarkers, the lack of overlap between biomarker sets correlated with MPR and ECV suggests that fibrosis and microvascular dysfunction may have distinct physiological and biochemical signatures. The associations between plasma biomarkers and CMR imaging markers observed in this study support the involvement of metabolic syndrome, renal disease, and systemic inflammation in the development of myocardial fibrosis and microvascular disease in HFpEF. Additional research is needed to delineate the relationships between biomarkers, imaging findings, and natural progression of HFpEF.

## Data Availability

The raw data supporting the conclusions of this article will be made available by the authors, without undue reservation.
