# Supplementary material for: Cardiometabolic biomarker patterns associated with cardiac MRI defined fibrosis and microvascular dysfunction in patients with heart failure with preserved ejection fraction
Source: Front Cardiovasc Med. 2024 Mar 4;11:1334226. doi: 10.3389/fcvm.2024.1334226 (PMC10945015; doi:10.3389/fcvm.2024.1334226)
Supplement: Supplementary file 1 [file Table1.docx]

Supplementary Table

| Name | Abbreviation | Corr with ECV | Corr with  MPR | Function** |
| --- | --- | --- | --- | --- |
| Angiogenin | ANG | 0.08519365 | -0.126962 | Binds to actin on the surface of endothelial cells; once bound, angiogenin is endocytosed and translocated to the nucleus. Angiogenin induces vascularization of normal and malignant tissues. |
| Angiopoietin-related protein 3 | ANGPTL3 | 0.05453344 | 0.065363 | Acts in part as a hepatokine that is involved in regulation of lipid and glucose metabolism |
| Membrane primary amine oxidase | AOC3 | -0.039334 | -0.0432951 | participates in lymphocyte extravasation and recirculation by mediating the binding of lymphocytes to peripheral lymph node vascular endothelial cells. May play a role in adipogenesis. |
| Apolipoprotein M | APOM | -0.1100468 | 0.17124358 | Involved in lipid transport. |
| Complement C1q tumor necrosis factor-related protein 1 | C1QTNF1 | 0.11368535 | -0.0663159 | Unknown Function |
| Complement C2 | C2 | 0.01629179 | -0.1075751 | Component C2 which is part of the classical pathway of the complement system |
| Carbonic Anhydrase 1 | CA1 | 0.02297065 | 0.02590118 | Reversible hydration of carbon dioxide. |
| Carbonic Anhydrase 3 | CA3 | 0.02890214 | 0.08512421 | Reversible hydration of carbon dioxide. |
| Carbonic Anhydrase 4 | CA4 | -0.1903837* | 0.12763428 | Reversible hydration of carbon dioxide. May stimulate the sodium/bicarbonate transporter activity of SLC4A4 that acts in pH homeostasis. |
| C-C motif chemokine 14 | CCL14 | 0.02604536 | -0.0053093 | induces intracellular Ca(2+) changes and enzyme release, but no chemotaxis, at concentrations of 100-1,000 nM, and is inactive on T-lymphocytes, neutrophils, and eosinophil leukocytes. Enhances the proliferation of CD34 myeloid progenitor cells. |
| C-C motif chemokine 18 | CCL18 | 0.16476681 | -0.1184497 | Chemotactic factor that attracts lymphocytes but not monocytes or granulocytes. May be involved in B-cell migration into B-cell follicles in lymph nodes. |
| C-C motif chemokine 5 | CCL5 | 0.063235 | -0.1311422 | Chemoattractant for blood monocytes, memory T-helper cells and eosinophils. Causes the release of histamine from basophils and activates eosinophils. By activating GPR75 may also play a role in insulin secretion by islet cells |
| Membrane cofactor protein | CD46 | 0.0234898 | -0.0711118 | Role in the complement system. Acts as a cofactor for complement factor I, a serine protease which protects autologous cells against complement-mediated injury by cleaving C3b and C4b deposited on host tissue. |
| CD59 glycoprotein | CD59 | -0.0575676 | 0.11038996 | Potent inhibitor of the complement membrane attack complex (MAC) action. |
| Cadherin-1 | CDH1 | 0.11506054 | -0.1192936 | CDH1 is involved in mechanisms regulating cell-cell adhesions, mobility and proliferation of epithelial cells |
| Liver carboxylesterase 1 | CES1 | -0.1603744 | 0.06590061 | Involved in the detoxification of xenobiotics and in the activation of ester and amide prodrugs. Hydrolyzes aromatic and aliphatic esters, but has no catalytic activity toward amides or a fatty acyl-CoA ester. |
| Complement factor H-related protein 5 | CFHR5 | 0.02580996 | -0.0570846 | Involved in complement regulation. The dimerized forms have avidity for tissue-bound complement fragments and efficiently compete with the physiological complement inhibitor CFH. |
| Neural cell adhesion molecule L1-like protein | CHL1 | -0.2219107* | 0.28194261* | Extracellular matrix and cell adhesion protein that plays a role in nervous system development and in synaptic plasticity. |
| Beta-Ala-His dipeptidase | CNDP1 | -0.2284996* | 0.11229453 | Catalyzes the peptide bond hydrolysis in Xaa-His dipeptides, displaying the highest activity toward carnosine and anserine |
| Collagen alpha-1(XVIII) chain | COL18A1 | 0.13103878 | -0.1412591 | May regulate extracellular matrix-dependent motility and morphogenesis of endothelial and non-endothelial cells. Endostatin: Potently inhibits endothelial cell proliferation and angiogenesis |
| Cartilage oligomeric matrix protein | COMP | -0.0929523 | 0.13429664 | May play a role in the structural integrity of cartilage via its interaction with other extracellular matrix proteins such as the collagens and fibronectin. Essential for maintaining a vascular smooth muscle cells (VSMCs) contractile/differentiated phenotype under physiological and pathological stimuli. |
| Complement receptor type 2 | CR2 | -0.0177437 | 0.00641031 | Receptor for complement C3, for the Epstein-Barr virus on human B-cells and T-cells and for HNRNPU. Participates in B lymphocytes activation |
| Cartilage acidic protein 1 | CRTAC1 | -0.0160383 | 0.00721206 | No functional description |
| Cystatin-C | CST3 | 0.09324448 | -0.0950582 | As an inhibitor of cysteine proteinases, this protein is thought to serve an important physiological role as a local regulator of this enzyme activity. |
| Neutrophil defensin 1 | DEFA1 | 0.03768807 | -0.1476774 | Defensin 1 and defensin 2 have antibacterial, fungicide and antiviral activities. Has antimicrobial activity against Gram-negative and Gram-positive bacteria. Defensins are thought to kill microbes by permeabilizing their plasma membrane. |
| Dipeptidyl Peptidase 4 | DPP4 | -0.2586159* | 0.10839171 | Cell surface glycoprotein receptor involved in the costimulatory signal essential for T-cell receptor (TCR)-mediated T-cell activation. |
| EGF-containing fibulin-like extracellular matrix protein 1 | EFEMP1 | 0.14592783 | 0.00284277 | Binds EGFR, the EGF receptor, inducing EGFR autophosphorylation and the activation of downstream signaling pathways. May play a role in cell adhesion and migration. |
| Endoglin | ENG | -0.0615491 | 0.13021735 | Vascular endothelium glycoprotein that plays an important role in the regulation of angiogenesis |
| Coagulation factor XI | F11 | -0.0659111 | 0.00356462 | Factor XI triggers the middle phase of the intrinsic pathway of blood coagulation by activating factor IX. |
| Coagulation factor VII | F7 | -0.1227333 | 0.11927084 | Initiates the extrinsic pathway of blood coagulation. Serine protease that circulates in the blood in a zymogen form. |
| Prolyl endopeptidase FAP | FAP | -0.0435947 | 0.02540172 | Cell surface glycoprotein serine protease that participates in extracellular matrix degradation and involved in many cellular processes including tissue remodeling, fibrosis, wound healing, inflammation and tumor growth. |
| Low affinity immunoglobulin gamma Fc region receptor II-a | FCGR2A | 0.06122775 | 0.02143937 | Binds to the Fc region of immunoglobulins gamma. Low affinity receptor. By binding to IgG it initiates cellular responses against pathogens and soluble antigens. |
| Low affinity immunoglobulin gamma Fc region receptor III-B | FCGR3B | -0.0536071 | 0.14417055 | Receptor for the Fc region of immunoglobulins gamma. Low affinity receptor. Binds complexed or aggregated IgG and also monomeric IgG. Contrary to III-A, is not capable to mediate antibody-dependent cytotoxicity and phagocytosis. |
| Ficolin-2 | FCN2 | -0.1001524 | 0.05018508 | May function in innate immunity through activation of the lectin complement pathway. |
| Fetuin B | FETUB | 0.03063595 | -0.1663136* | Protease inhibitor required for egg fertilization. |
| Growth arrest-specific protein 6 | GAS6 | 0.03731342 | 0.06812912 | Ligand for tyrosine-protein kinase receptors AXL, TYRO3 and MER whose signaling is implicated in cell growth and survival, cell adhesion and cell migration. |
| Granulysin | GNLY | 0.02840044 | -0.061468 | Antimicrobial protein that kills intracellular pathogens. Active against a broad range of microbes, including Gram-positive and Gram-negative bacteria, fungi, and parasites. Kills Mycobacterium tuberculosis. |
| Platelet glycoprotein Ib alpha chain | GP1BA | -0.0639968 | -0.0517124 | GP-Ib, a surface membrane protein of platelets, participates in the formation of platelet plugs by binding to the A1 domain of vWF, which is already bound to the subendothelium. |
| Intercellular adhesion molecule 1 | ICAM1 | -0.0017045 | -0.1283478 | ICAM proteins are ligands for the leukocyte adhesion protein LFA-1 (integrin alpha-L/beta-2). During leukocyte trans-endothelial migration, ICAM1 engagement promotes the assembly of endothelial apical cups through ARHGEF26/SGEF and RHOG activation. |
| Intercellular adhesion molecule 3 | ICAM3 | 0.00028859 | 0.09332953 | ICAM proteins are ligands for the leukocyte adhesion protein LFA-1 (integrin alpha-L/beta-2) ICAM3 is also a ligand for integrin alpha-D/beta-2. In association with integrin alpha-L/beta-2, contributes to apoptotic neutrophil phagocytosis by macrophages |
| Insulin-like growth factor-binding protein 3 | IGFBP3 | -0.0239148 | 0.0492858 | IGF-binding proteins prolong the half-life of the IGFs and have been shown to either inhibit or stimulate the growth promoting effects of the IGFs on cell culture. They alter the interaction of IGFs with their cell surface receptors. Also exhibits IGF-independent antiproliferative and apoptotic effects mediated by its receptor TMEM219/IGFBP-3R. |
| Insulin-like growth factor-binding protein 6 | IGFBP6 | -0.0875753 | 0.10000578 | IGF-binding proteins prolong the half-life of the IGFs and have been shown to either inhibit or stimulate the growth promoting effects of the IGFs on cell culture. They alter the interaction of IGFs with their cell surface receptors. |
| Immunoglobulin lambda constant 2 | IGLC2 | 0.16088498 | -0.0646916 | Constant region of immunoglobulin light chains. Immunoglobulins, also known as antibodies, are membrane-bound or secreted glycoproteins produced by B lymphocytes. In the recognition phase of humoral immunity, the membrane-bound immunoglobulins serve as receptors which, upon binding of a specific antigen, trigger the clonal expansion and differentiation of B lymphocytes into immunoglobulins-secreting plasma cells. |
| Interleukin-7 receptor subunit alpha | IL7R | -0.0788632 | 0.17396861 | Receptor for interleukin-7. Also acts as a receptor for thymic stromal lymphopoietin (TSLP). |
| Integrin alpha-M | ITGAM | 0.01344845 | 0.00186175 | Integrin ITGAM/ITGB2 is implicated in various adhesive interactions of monocytes, macrophages and granulocytes as well as in mediating the uptake of complement-coated particles and pathogens |
| Mast/stem cell growth factor receptor Kit | KIT | -0.2078061* | 0.15309794 | Tyrosine-protein kinase that acts as cell-surface receptor for the cytokine KITLG/SCF and plays an essential role in the regulation of cell survival and proliferation, hematopoiesis, stem cell maintenance, gametogenesis, mast cell development, migration and function, and in melanogenesis. |
| Neutrophil gelatinase-associated lipocalin | LCN2 | -0.0163602 | -0.0140449 | Iron-trafficking protein involved in multiple processes such as apoptosis, innate immunity and renal development |
| Leukocyte immunoglobulin-like receptor subfamily B member 1 | LILRB1 | -0.0010453 | -0.0473934 | Engagement of LILRB1 present on natural killer cells or T-cells by class I MHC molecules protects the target cells from lysis. Interaction with HLA-B or HLA-E leads to inhibition of FCER1A signaling and serotonin release |
| Leukocyte immunoglobulin-like receptor subfamily B member 2 | LILRB2 | -0.0367852 | -0.0770396 | Receptor for class I MHC antigens. Recognizes a broad spectrum of HLA-A, HLA-B, HLA-C, HLA-G and HLA-F alleles. Involved in the down-regulation of the immune response and the development of tolerance. |
| Leukocyte immunoglobulin-like receptor subfamily B member 5 | LILRB5 | 0.11862506 | 0.04461537 | May act as receptor for class I MHC antigens. |
| Latent-transforming growth factor beta-binding protein 2 | LTBP2 | 0.03324567 | 0.06978173 | May play an integral structural role in elastic-fiber architectural organization and/or assembly. |
| Lymphatic vessel endothelial hyaluronic acid receptor 1 | LYVE1 | -0.1213298 | 0.14395459 | Ligand-specific transporter trafficking between intracellular organelles (TGN) and the plasma membrane. Plays a role in autocrine regulation of cell growth mediated by growth regulators containing cell surface retention sequence binding (CRS). |
| Mannose-binding protein C | MBL2 | -0.020082 | 0.10123194 | Calcium-dependent lectin involved in innate immune defense. Binds mannose, fucose and N-acetylglucosamine on different microorganisms and activates the lectin complement pathway. |
| Multiple epidermal growth factor-like domains protein 9 | MEGF9 | -0.2343378* | 0.13154203 | no UniProt functional description available |
| Hepatocyte growth factor receptor | MET | -0.0487822 | 0.03372581 | Receptor tyrosine kinase that transduces signals from the extracellular matrix into the cytoplasm by binding to hepatocyte growth factor/HGF ligand. Regulates many physiological processes including proliferation, scattering, morphogenesis and survival. |
| Microfibrillar-associated protein 5 | MFAP5 | -0.035456 | 0.02886522 | In the cardiovascular system, could regulate growth factors or participate in cell signaling in maintaining large vessel integrity |
| Neural Cell Adhesion Molecule 1 | NCAM1 | -0.1643093 | 0.25026527* | This protein is a cell adhesion molecule involved in neuron-neuron adhesion, neurite fasciculation, outgrowth of neurites, etc. |
| Nidogen-1 | NID1 | 0.03970448 | -0.0278466 | Sulfated glycoprotein widely distributed in basement membranes and tightly associated with laminin. Also binds to collagen IV and perlecan. It probably has a role in cell-extracellular matrix interactions. |
| Neurogenic locus notch homolog protein 1 | NOTCH1 | -0.0699714 | 0.13147126 | Affects the implementation of differentiation, proliferation and apoptotic programs. Involved in angiogenesis; negatively regulates endothelial cell proliferation and migration and angiogenic sprouting. |
| Neuropilin-1 | NRP1 | 0.0540007 | 0.01274854 | Receptor involved in the development of the cardiovascular system, in angiogenesis, in the formation of certain neuronal circuits and in organogenesis outside the nervous system. |
| Oncostatin-M-specific receptor subunit beta | OSMR | 0.01057016 | 0.1606317 | Associates with IL31RA to form the IL31 receptor. Binds IL31 to activate STAT3 and possibly STAT1 and STAT5. Capable of transducing OSM-specific signaling events |
| Peptidyl-glycine alpha-amidating monooxygenase | PAM | 0.05010472 | -0.0215009 | Bifunctional enzyme that catalyzes the post-translational modification of inactive peptidylglycine precursors to the corresponding bioactive alpha-amidated peptides, a terminal modification in biosynthesis of many neural and endocrine peptides |
| Procollagen C-endopeptidase enhancer 1 | PCOLCE | 0.16299043 | -0.1849193* | Binds to the C-terminal propeptide of type I procollagen and enhances procollagen C-proteinase activity.  C-terminal processed part of PCPE (CT-PCPE) may have an metalloproteinase inhibitory activity. |
| Platelet-activating factor acetylhydrolase | PLA2G7 | -0.1439063 | 0.16411039* | Modulates the action of platelet-activating factor (PAF) by hydrolyzing the sn-2 ester bond to yield the biologically inactive lyso-PAF. Has a specificity for substrates with a short residue at the sn-2 position. It is inactive against long-chain phospholipids. |
| Phospholipid transfer protein | PLTP | 0.15336979 | -0.0144988 | Essential for the transfer of excess surface lipids from triglyceride-rich lipoproteins to HDL, thereby facilitating the formation of smaller lipoprotein remnants, contributing to the formation of LDL, and assisting in the maturation of HDL particles. PLTP also plays a key role in the uptake of cholesterol from peripheral cells and tissues that is subsequently transported to the liver for degradation and excretion. |
| Plexin-B2 | PLXNB2 | 0.05822867 | -0.0880738 | Plays a role in glutamatergic synapse development and is required for SEMA4A-mediated excitatory synapse development (By similarity). Binding to class 4 semaphorins promotes downstream activation of RHOA and phosphorylation of ERBB2 at 'Tyr-1248' (By similarity). Required for normal differentiation and migration of neuronal cells during brain corticogenesis and for normal embryonic brain development (By similarity). |
| Lysosomal Pro-X carboxypeptidase | PRCP | -0.071141 | -0.0112907 | Cleaves C-terminal amino acids linked to proline in peptides such as angiotensin II, III and des-Arg9-bradykinin. This cleavage occurs at acidic pH, but enzymatic activity is retained with some substrates at neutral pH. |
| Vitamin K-dependent protein C | PROC | -0.0207948 | -0.0040245 | Protein C is a vitamin K-dependent serine protease that regulates blood coagulation by inactivating factors Va and VIIIa in the presence of calcium ions and phospholipids Exerts a protective effect on the endothelial cell barrier function |
| Trypsin-2 | PRSS2 | 0.03969955 | -0.1010911 | in the ileum, may be involved in defensin processing, including DEFA5. |
| Receptor-type tyrosine-protein phosphatase S | PTPRS | -0.1228235 | 0.13268308 | Binding to chondroitin sulfate and heparan sulfate proteoglycans has opposite effects on PTPRS oligomerization and regulation of neurite outgrowth. Contributes to the inhibition of neurite and axonal outgrowth by chondroitin sulfate proteoglycans, also after nerve transection. Plays a role in stimulating neurite outgrowth in response to the heparan sulfate proteoglycan GPC2 |
| Glutaminyl-peptide cyclotransferase | QPCT | -0.0868217 | 0.14882684 | Responsible for the biosynthesis of pyroglutamyl peptides.  May be involved in the N-terminal pyroglutamate formation of several amyloid-related plaque-forming peptides. |
| Lithostathine-1-alpha | REG1A | 0.19905939 | -0.144338 | Might act as an inhibitor of spontaneous calcium carbonate precipitation. May be associated with neuronal sprouting in brain, and with brain and pancreas regeneration. |
| Regenerating islet-derived protein 3-alpha | REG3A | 0.04743026 | 0.0705231 | Bactericidal C-type lectin which acts exclusively against Gram-positive bacteria and mediates bacterial killing by binding to surface-exposed carbohydrate moieties of peptidoglycan. Regulates keratinocyte proliferation and differentiation after skin injury via activation of EXTL3-PI3K-AKT signaling pathway. |
| Serum amyloid A-4 protein | SAA4 | 0.10582671 | -0.1034569 | Major acute phase reactant. |
| L-selectin | SELL | -0.136321 | 0.06970208 | Mediates the adherence of lymphocytes to endothelial cells of high endothelial venules in peripheral lymph nodes. |
| Plasma serine protease inhibitor | SERPINA5 | -0.0718467 | -0.0360907 | Plays hemostatic roles in the blood plasma. Acts as a procoagulant and proinflammatory factor by inhibiting the anticoagulant activated protein C factor as well as the generation of activated protein C factor by the thrombin/thrombomodulin complex. Acts as an anticoagulant factor by inhibiting blood coagulation factors like prothrombin, factor XI, factor Xa, plasma kallikrein and fibrinolytic enzymes such as tissue- and urinary-type plasminogen activators. |
| Thyroxine-binding globulin | SERPINA7 | -0.1818163 | -0.0110882 | Major thyroid hormone transport protein in serum. |
| Superoxide dismutase [Cu-Zn] | SOD1 | -0.0078521 | 0.03177046 | Destroys radicals which are normally produced within the cells and which are toxic to biological systems. |
| SPARC-like protein 1 | SPARCL1 | 0.05279221 | 0.08149058 | There is no UniProt functional description available for this protein at present |
| Beta-galactoside alpha-2,6-sialyltransferase 1 | ST6GAL1 | 0.07741879 | -0.0943887 | Transfers sialic acid from CMP-sialic acid to galactose-containing acceptor substrates. |
| Transcobalamin-2 | TCN2 | 0.14806031 | -0.0461886 | Primary vitamin B12-binding and transport protein. Delivers cobalamin to cells. |
| Transforming growth factor-beta-induced protein ig-h3 | TGFBI | 0.04583775 | 0.02833545 | Plays a role in cell adhesion (PubMed:8024701). May play a role in cell-collagen interactions (By similarity). |
| Transforming growth factor beta receptor type 3 | TGFBR3 | 0.08005102 | 0.09792883 | Binds to TGF-beta. Could be involved in capturing and retaining TGF-beta for presentation to the signaling receptors. |
| Thrombospondin-4 | THBS4 | -0.0213161 | 0.08870122 | Binds to structural extracellular matrix (ECM) proteins and modulates the ECM in response to tissue damage, contributing to cardioprotective and adaptive ECM remodeling. |
| Tyrosine-protein kinase receptor Tie-1 | TIE1 | -0.0372729 | 0.03057293 | Transmembrane tyrosine-protein kinase that may modulate TEK/TIE2 activity and contribute to the regulation of angiogenesis. |
| T-cell immunoglobulin and mucin domain-containing protein 4 | TIMD4 | 0.14873753 | -0.1554057* | Phosphatidylserine receptor that enhances the engulfment of apoptotic cells. Involved in regulating T-cell proliferation and lymphotoxin signaling. |
| Metalloproteinase inhibitor 1 | TIMP1 | 0.14889455 | -0.1282272 | Metalloproteinase inhibitor that functions by forming one to one complexes with target metalloproteinases, such as collagenases, and irreversibly inactivates them by binding to their catalytic zinc cofactor.  Also functions as a growth factor that regulates cell differentiation, migration and cell death and activates cellular signaling cascades via CD63 and ITGB1. |
| Tenascin | TNC | 0.06951911 | 0.10927727 | Extracellular matrix protein implicated in guidance of migrating neurons as well as axons during development, synaptic plasticity as well as neuronal regeneration. Promotes neurite outgrowth from cortical neurons grown on a monolayer of astrocytes. |
| Tenascin-X | TNXB | -0.0227728 | 0.04784193 | Appears to mediate interactions between cells and the extracellular matrix. Substrate-adhesion molecule that appears to inhibit cell migration. Accelerates collagen fibril formation. |
| Uromodulin | UMOD | -0.184569* | 0.16227185 | Functions in biogenesis and organization of the apical membrane of epithelial cells of the thick ascending limb of Henle's loop (TALH), where it promotes formation of complex filamentous gel-like structure that may play a role in the water barrier permeability |
| Vasorin | VASN | -0.1660589 | 0.04570854 | May act as an inhibitor of TGF-beta signaling. |
| Vascular cell adhesion protein 1 | VCAM1 | 0.07006702 | -0.0477567 | Important in cell-cell recognition. Appears to function in leukocyte-endothelial cell adhesion. The VCAM1/ITGA4/ITGB1 interaction may play a pathophysiologic role both in immune responses and in leukocyte emigration to sites of inflammation. |

*Significant Correlation Coefficients

**All function descriptions are obtained from the OLINK website
